# Achieving Representation in Cardiovascular Device Clinical Research: The Role of Regulatory Science

**DOI:** 10.1016/j.jscai.2026.104255

**Published:** 2026-02-12

**Authors:** Wayne B. Batchelor, Megan Coylewright, Yasser Jamil, Roseann White, Ernest Spitzer, Nada Hanafi, Michael R. Jaff, Roberta Chapman, Dan Stephens, Mitchell W. Krucoff

**Affiliations:** a Medicine and Schar Heart and Vascular Service Lines, Inova Health System, Fairfax, Virginia; b Heart and Vascular Center, Essentia Health, Duluth, Minnesota; c Your 3rd Opinion, Chapel Hill, North Carolina; d European Cardiovascular Research Institute, Rotterdam, the Netherlands; e Cardialysis, Rotterdam, the Netherlands; f Medtech Strategy Advisors, LLC, San Jose, California; g Boston Scientific Corporation, Marlborough, Massachusetts; h Johnson & Johnson MedTech, New Brunswick, New Jersey; i Boston Scientific Corporation, Maple Grove, Minnesota; j Division of Cardiology, Department of Medicine, Duke University Medical Center, Durham, North Carolina

**Keywords:** cardiovascular devices, diversity action plan, representation

## Introduction

**Figure 1:**
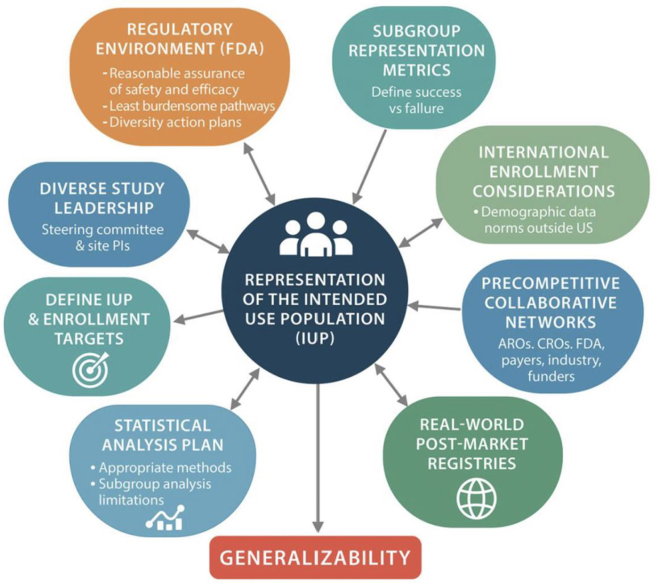
**Key determinants influencing representation of the intended-use population in clinical trials and generalizability.** ARO, academic research organization; CRO, clinical research organization; FDA, US Food and Drug Administration; PI, principal investigator.

Cardiovascular device therapies, such as transcatheter aortic valve replacement and implantable cardioverter-defibrillators, have revolutionized the treatment of advanced heart disease, often offering improvements in quality of life and survival. Generally considered class III (high risk), most cardiovascular devices require rigorous US Food and Drug Administration (FDA) premarket approval (PMA) to ensure safety and effectiveness. However, the data that underpin the assessment of risk versus benefit and device approval are often not representative of the intended-use population (IUP). To date, women, demographic minorities, older adults, patients living in rural areas, and low-income households have been underrepresented in PMA studies, undermining generalizability.^1^^,^^2^ In this viewpoint, we explore how improving representation in cardiovascular device research studies aligns with the principles of regulatory science and discuss the challenges of achieving this goal. A visual summary of the article’s core concepts is presented in Figure 1.

## Regulatory science as a foundation for representation in clinical research

Regulatory science includes the scientific tools, standards, and methodologies used to assess the safety, effectiveness, and quality of medical products. It plays a vital role in ensuring that clinical trials are representative of a device’s IUP while accounting for key statutory requirements, statistical considerations, and the broader research ecosystem (ie, device manufacturers, investigators, research organizations, health systems, patients, payors, and regulators). Although a strong regulatory science framework is essential to promoting representation and improving the generalizability of study results, there are several statutory requirements that serve as boundaries within which regulatory science must function.

## Statutory requirements governing class III device research

The FDA’s authority in regulating class III cardiovascular devices is anchored by the following 3 key statutory provisions:

1. Reasonable assurance of safety and effectiveness (Federal Food, Drug, and Cosmetic [FD&C] Act, Section 520e, 1976): this grants the FDA authority to limit the sale, distribution, or use of a device without sufficient evidence of benefit-risk balance—the net benefits of a device must be balanced with its associated risks.^3^
2. Least burdensome pathways (Food and Drug Administration Modernization Act [FDAMA], 1997): this seeks to streamline data collection and minimize unnecessary burdens that could delay new device approval, without compromising existing standards for device safety and effectiveness.^4^
3. Diversity action plans (Food and Drug Omnibus Reform Act [FDORA], 2022): this mandates the submission of diversity action plans (DAPs) to the FDA for all investigational device exemption (IDE) studies to ensure the representation of the IUP in pivotal studies, including adequate enrollment from underrepresented populations.^5^

These statutory requirements set the regulatory boundaries for cardiovascular device trials, including measures that can be taken to address the pervasive underrepresentation of demographic groups that lead to suboptimal representation of the IUP in pivotal clinical trials.

## Role of precompetitive collaborative communities

As regulators, industry partners, investigators, and other stakeholders begin implementing the FDORA requirements mandating DAPs, precompetitive collaborative communities offer a unique opportunity to develop consensus on strategies to improve representation of the IUP. Several established communities, including the Medical Device Epidemiology Network, the Heart Valve Collaboratory, the Heart Failure Collaboratory, the Academic Research Consortium, and the Cardiac Safety Research Consortium, are particularly well positioned to lead these efforts. Each has a demonstrated history of convening regulators, clinicians, academicians, and industry to address methodological and operational challenges in cardiovascular device trials.

Recent federal executive orders have terminated all government diversity, equity, and inclusion programs and led to the temporary removal of the FDA’s draft guidance on DAPs. As a result, sponsors face uncertainty around enrollment targets, evidentiary requirements, and the scope of permissible diversity-focused strategies. This ambiguity has created practical challenges in constructing DAPs that better reflect the IUP. These challenges are further compounded by increased legal scrutiny of demographically conscious enrollment approaches and heightened institutional caution among government-facing officials regarding the pace and parameters of diversity initiatives. Precompetitive collaborative communities have the benefit of operating within a politically insulated yet scientifically authoritative environment. This makes them useful platforms to advance best practices for the development and implementation of DAPs.

Given the current environment, the following near-term actions can help sustain momentum and provide clearer direction for sponsors and regulators:

1. Developing standardized DAP templates grounded in disease epidemiology and regulatory science principles.
2. Sharing datasets and validated analytic tools that support trial enrichment strategies, feasibility assessments, and representation forecasts.
3. Defining “safe harbor” practices including geography-based site selection, use of socioeconomic proxies, and prespecified subgroup enrollment strategies that enhance representation.

Collectively, these strategies can strengthen the scientific foundation for DAPs, reduce uncertainty for trial sponsors, and advance more equitable and generalizable evidence generation from cardiovascular device trials.

## Challenges in designing and implementing DAPs

### Defining the IUP and subgroup targets

DAPs seek to align clinical trial enrollment with the demographics of the IUP. However, accurately defining the IUP may be challenging, since variations in disease burden, diagnostic accuracy, and referral and treatment biases can obscure its measurement.^6^ For example, real-world device registries tend to underrepresent marginalized groups because of poor access to primary care, lower rates of diagnostic testing and referral, and other systemic biases. A standardized approach to determining the appropriate demographic mix of representative patient cohorts enrolled in PMA studies remains an area of continued investigation that will require multistakeholder consensus.

### Statistical considerations

Beyond planning for primary efficacy and safety end points, statistical planning in IDE studies may also help determine whether the enrolled population adequately reflects the IUP and whether meaningful differences in safety or effectiveness exist across key subgroups. These latter objectives may require distinct sample-size calculations and statistical analytic plans that should not be conflated with those used for primary end point analysis. The participation-to-prevalence ratio (PPR) is an established tool for quantifying the degree to which the enrolled subgroups reflect their prevalence in the disease population. For example, to measure the adequacy of inclusion of Black study subjects in a cardiovascular device trial, PPR would be calculated as:

$$\text{P}\text{P}\text{R}=\frac{\%\;\text{B}\text{l}\text{a}\text{c}\text{k}\;\text{s}\text{t}\text{u}\text{d}\text{y}\;\text{s}\text{u}\text{b}\text{j}\text{e}\text{c}\text{t}\text{s}\;\text{e}\text{n}\text{r}\text{o}\text{l}\text{l}\text{e}\text{d}}{\%\;\text{Black}\;\text{patients}\;\text{in}\;\text{the}\;\text{IUP}}$$

A PPR <0.8 typically indicates underrepresentation, 0.8–1.2 suggests adequate alignment, and >1.2 denotes overrepresentation.^7^

The utility of this metric depends on having a reliable estimate of disease prevalence within subgroups (ie, denominator of the above equation), which is often lacking—such as is the case for aortic valve disease in Black patients. Furthermore, most IDE studies designed to support PMA-device applications to the FDA are statistically powered to detect the treatment effects in the overall study population and not within individual subgroups.^1^^,^^6^ To achieve adequate statistical power to evaluate safety and effectiveness end points in all pertinent subgroups would drastically increase trial size, duration, and cost—contradicting the “least burdensome” statutory principle. Therefore, although optimal representation of the IUP should be strived for within IDE studies, other solutions may be necessary to garner insight into outcomes for demographic subgroups, such as supplementing with postmarket real-world evidence registries to increase precision around the point estimates for key safety and effectiveness end points.

Although larger sample sizes may be necessary to ensure meaningful subgroup representation, such investments may be justified when balanced against the potential risks of inadequate representation. Insufficient enrollment can delay or obscure detection of subgroup-specific safety signals or differential treatment effects, contribute to inequitable postapproval diffusion of effective therapies, and increase the likelihood that serious adverse events emerge after widespread clinical adoption. These downstream consequences, and the accompanying erosion of public trust, underscore why proactive statistical planning for relevant subgroup analyses may be methodologically prudent.

### Defining success with DAPs: how good is good enough?

Assessing whether a DAP is “successful” remains another key challenge. Several critical questions emerge: (1) Should success be defined by the PPR, a prespecified numeric enrollment target, epidemiologic benchmarks, population demographics, or another metric? (2) How should inadequate subgroup enrollment be defined, and what corrective actions should be taken if enrollment targets are not met? and (3) Is the overrepresentation of a subgroup problematic? Addressing these questions requires harmonized, consensus-driven standards and metrics to guide the implementation and accountability of DAPs.

Until a universally accepted benchmark for “ideal” representation is established, prolonged debate without meaningful action risks perpetuating longstanding patterns of underrepresentation. Therefore, pivotal IDE trials should aim for PPRs ≥0.8 for prespecified clinically relevant demographic subgroups. This target can be strengthened by incorporating complementary metrics, such as subgroup enrollment percentages or prespecified minimum absolute enrollment numbers, to ensure that representation is sufficient to support valid inference.

### Generalizability of results

The generalizability of IDE trial results depends not only on robust end points and statistical analysis plans but also on aligning the study sample with the IUP. Underrepresentation of women, racial and ethnic minorities, older adults, rural populations, and individuals from low-income households—often driven by an overreliance on academic medical centers rather than community-based sites—can undermine the external validity of device trials. In such cases, regulators may consider conditional approval pending supplemental data that confirms safety and efficacy in more representative patient cohorts.

### Use of postmarket device registries

Regulatory agencies, including the Centers for Medicare and Medicaid Services, are increasingly mandating postmarket registries to supplement IDE findings and ensure continued evidence development for newly approved devices entering the commercial market. These registries may be directly run by device manufacturers or through professional societies such as the STS/ACC TVT Registry.^8^ The primary advantages of these registries include: (1) continued monitoring of device performance and safety following FDA approval; (2) tracking patient demographics in the commercial setting; and (3) evaluation of longer-term outcomes. If widespread commercial device use raises subgroup-specific safety or efficacy concerns, postmarket “course corrections,” including labeling revisions, indication restrictions, targeted safety reviews, and enhanced adverse-event monitoring, may be necessary. The lack of enforcement of DAPs may increase reliance on these corrective measures at a time when federal expectations for premarket representation remain uncertain. However, these postmarket approaches may be limited by observational study designs, incomplete capture of social and structural determinants of health, and the potential for persistent treatment biases. Consequently, inequities in outcomes may be detected only after patients have already been exposed to avoidable harm and public trust potentially compromised. For these reasons, ensuring robust representation of the IUP within pivotal, randomized IDE trials must remain the goal. Postmarket surveillance may complement but should not supplant the foundational role of representative PMA randomized device trials.

### International considerations

Global device trials and DAPs must reconcile varying national regulatory norms. Although many underrepresented populations, such as women, older adults, rural patients, and individuals from low-income households, are relevant across global study settings, the definition of demographic minorities varies by country. For example, most European countries, except the United Kingdom and Ireland, classify demographic groups based on ethnicity, country of birth, ancestry, or language rather than race. The collection of race and ethnicity data in Europe is uncommon, largely because of stringent antidiscrimination and data privacy regulations.^9^ An emerging approach is to incorporate census-defined demographic categories, which enable the identification of locally underrepresented populations. However, integrating such data into US–based regulatory submissions—while still meeting the objectives of DAPs—remains a challenge. Addressing this issue will require coordinated, multinational collaboration to harmonize definitions and data standards across international jurisdictions.

## Conclusions

Ensuring the safety and effectiveness of class III cardiovascular devices requires a careful balance between fulfilling statutory regulatory requirements and achieving representative enrollment of the IUP in pivotal PMA studies. The inclusion of historically underrepresented populations hinges on pragmatic, adaptive, and inclusive study designs; accountable and diverse executive leadership; strategic site selection; rigorous statistical planning; and the implementation of a thoughtfully constructed DAP. Although real-world postmarket approval registries may complement IDE trial data by refining point estimates of safety and effectiveness and evaluating device performance in broader populations, representation of the IUP within pivotal PMA trials must remain a central goal. The absence of clearly defined standards and best practices for DAPs remains a critical barrier to progress—one that can only be overcome through sustained, multistakeholder collaboration grounded in rigorous regulatory science.
